# Supplementary material for: Spatial aggregation with respect to a population distribution: Impact on inference
Source: Spat Stat. Author manuscript; Available in PMC 2024 Oct 31. (PMC11526805; doi:10.1016/j.spasta.2022.100714)
Supplement: Supplement [file NIHMS2029774-supplement-Supplement.pdf]

Supplementary Materials for "Spatial Aggregation with Respect to a Population Distribution"

John Paige[a,*], Geir-Arne Fuglstad[a], Andrea Riebler[a], Jon Wakefield[b]

[a] *Department of Mathematical Sciences, NTNU, Trondheim, Norway*
[b] *Department of Statistics and Biostatistics, University of Washington, Seattle, USA*

S.1. Impact of incorrect aggregation weights on MSE

Here we will examine the effect of using incorrect aggregation weights has on estimates of population averages and totals given a quantity of interest, $Y \sim \text{GP}(\mu(\cdot), C(\cdot, \cdot))$, spatial mean function $\mu(\cdot)$, and spatial covariance function $C(\cdot, \cdot)$. If the estimated (or assumed) population density is $\hat{q}$, the estimated population total (or average) for region R is,

$$Y(R, \hat{q}) = \int_R \hat{q}(\boldsymbol{s})Y(\boldsymbol{s}) \, \mathrm{d}\boldsymbol{s}, \tag{A.1}$$

where $\hat{q}$ is normalized to have unit integral in region R when estimating an average.

We derive the mean square error (MSE) of (A.1) with respect to the true population average $Y(R, q)$ for true population density q in the supplement in Section S.1. For $\hat{q}$ being the estimated/assumed population density, and Y defined as above, we find the MSE of our estimate for the population total in region R is:

$$\begin{aligned}
\text{MSE}(Y(R, \hat{q})) &= E\left[\left(\int_R \hat{q}(\boldsymbol{s})\mu(\boldsymbol{s}) \, \mathrm{d}\boldsymbol{s} - \int_R q(\boldsymbol{s})\mu(\boldsymbol{s}) \, \mathrm{d}\boldsymbol{s}\right)^2\right] \\
&= \left(\int_R (\hat{q} - q)(\boldsymbol{s})\mu(\boldsymbol{s}) \, \mathrm{d}\boldsymbol{s}\right)^2 - \int_R \int_R (\hat{q} - q)(\boldsymbol{s})(\hat{q} - q)(\boldsymbol{t})C(\boldsymbol{s} - \boldsymbol{t}) \, \mathrm{d}\boldsymbol{s} \, \mathrm{d}\boldsymbol{t} \\
&= \text{Bias}^2(Y(R, \hat{q}), Y(R, q)) + \text{Var}(Y(R, \hat{q} - q)).
\end{aligned}$$

If we assume that R is sufficiently small, so that $Y(\boldsymbol{s}) = \mu(\boldsymbol{s}) + \epsilon$ for $\boldsymbol{s} \in R$, where $\epsilon \sim N(0, \sigma^2)$, and if we make the further assumption that μ takes two different values, μ_1 for $\boldsymbol{s} \in A$ for $A \subset R$ and μ_2 for $\boldsymbol{s} \in R \setminus A$ where '\' denotes the 'set minus' operator. We will assume that $\int_A q(\boldsymbol{s}) \, \mathrm{d}\boldsymbol{s} = q_1$, and $\int_R q(\boldsymbol{s}) \, \mathrm{d}\boldsymbol{s} = T$, so that $\int_{R \setminus A} q(\boldsymbol{s}) \, \mathrm{d}\boldsymbol{s} = q_2 = T - q_1$. We similarly assume that the integral of $\hat{q}$ over A, R,

---

*Corresponding author
Email address: john.paige@ntnu.edu (John Paige)

and $R \setminus A$ is $\hat{q}_1$, $\hat{T}$, and $\hat{q}_2 = \hat{T} - \hat{q}_1$ respectively. The resulting bias is:

$$\begin{aligned}\text{Bias}(Y(R,\hat{q}), Y(R,q)) &= \int_R (\hat{q} - q)(\boldsymbol{s})\mu(\boldsymbol{s})\ \mathrm{d}\boldsymbol{s} \\ &= (\hat{q}_1 - q_1)\mu_1 + (\hat{q}_2 - q_2)\mu_2 \\ &= (\hat{q}_1 - q_1)\mu_1 + (\hat{T} - T + q_1 - \hat{q}_1)\mu_2 \\ &= (\hat{q}_1 - q_1)(\mu_1 - \mu_2) + (\hat{T} - T)\mu_2.\end{aligned}$$

The variance is:

$$\begin{aligned}\text{Var}(Y(R, \hat{q} - q)) &= \int_R \int_R (\hat{q} - q)(\boldsymbol{s})(\hat{q} - q)(\boldsymbol{t}) C(\boldsymbol{s} - \boldsymbol{t})\ \mathrm{d}\boldsymbol{s}\ \mathrm{d}\boldsymbol{t} \\ &= \sigma^2(\hat{q}_1 q_1 + \hat{q}_1(T - q_1) + (\hat{T} - \hat{q}_1)q_1 + (\hat{T} - \hat{q}_1)(T - q_1)) \\ &= \sigma^2 T\hat{T}.\end{aligned}$$

Hence, the MSE is:

$$\begin{aligned}\text{MSE}(Y(R,\hat{q})) &= ((\hat{q}_1 - q_1)\mu_1 + (\hat{q}_2 - q_2)\mu_2)^2 + \sigma^2 T\hat{T} \\ &= (\hat{q}_1 - q_1)^2(\mu_1 - \mu_2)^2 + 2(\hat{q}_1 - q_1)(\mu_1 - \mu_2)(\hat{T} - T)\mu_2 + (\hat{T} - T)^2\mu_2^2 + \sigma^2 T\hat{T}.\end{aligned}$$

If $\hat{T} = T$, such as in the case where q and $\hat{q}$ are both normalized to 1 (for example, when estimating risk or prevalence), then the MSE simplifies to:

$$\text{MSE}(Y(R,\hat{q})) = (\hat{q}_1 - q_1)^2(\mu_1 - \mu_2)^2 + \sigma^2 T^2.$$

If we assume that R is sufficiently small, so that $Y(\boldsymbol{s}) = \mu(\boldsymbol{s}) + \epsilon$ for $\boldsymbol{s} \in R$, where $\epsilon \sim N(0, \sigma^2)$, and if we make the further assumption that μ takes two different values, μ_1 for $\boldsymbol{s} \in A$ for $A \subset R$ and μ_2 for $\boldsymbol{s} \in R \setminus A$ where '$\setminus$' denotes the 'set minus' operator. We will assume that $\int_A q(\boldsymbol{s})\ \mathrm{d}\boldsymbol{s} = q_1$, and $\int_{R\setminus A} q(\boldsymbol{s})\ \mathrm{d}\boldsymbol{s} = q_2$. We similarly assume that the integral of $\hat{q}$ over A and $R \setminus A$ is $\hat{q}_1$ and $\hat{q}_2$ respectively. The resulting bias is:

$$\begin{aligned}\text{Bias}(Y(R,\hat{q}), Y(R,q)) &= \int_R (\hat{q} - q)(\boldsymbol{s})\mu(\boldsymbol{s})\ \mathrm{d}\boldsymbol{s} \\ &= (\hat{q}_1 - q_1)\mu_1 + (\hat{q}_2 - q_2)\mu_2.\end{aligned}$$

The variance is:

$$\begin{aligned}\text{Var}(Y(R, \hat{q} - q)) &= \int_R \int_R (\hat{q} - q)(\boldsymbol{s})(\hat{q} - q)(\boldsymbol{t}) C(\boldsymbol{s} - \boldsymbol{t})\ \mathrm{d}\boldsymbol{s}\ \mathrm{d}\boldsymbol{t} \\ &= \sigma^2(\hat{q}_1 q_1 + \hat{q}_1 q_2 + \hat{q}_2 q_1 + \hat{q}_2 q_2) \\ &= \sigma^2 T\hat{T}\end{aligned}$$

for $T = q_1 + q_2$ and $\hat{T} = \hat{q}_1 + \hat{q}_2$. Hence, the MSE is:

$$\begin{aligned}\text{MSE}(Y(R,\hat{q})) &= ((\hat{q}_1 - q_1)\mu_1 + (\hat{q}_2 - q_2)\mu_2)^2 + \sigma^2 T\hat{T} \\ &= (\hat{q}_1 - q_1)^2\mu_1^2 + 2(\hat{q}_1 - q_1)(\hat{q}_2 - q_2)\mu_1\mu_2 + (\hat{q}_2 - q_2)^2\mu_2^2 + \sigma^2 T\hat{T}.\end{aligned}$$

If $\hat{q}_1$ and $\hat{q}_2$ are random with $E[\hat{q}_1] = q_1 + b_1$ and $E[\hat{q}_2] = q_2 + b_2$ with $\mathrm{Var}(\hat{q}_1) = \mathrm{Var}(\hat{q}_2) = \nu^2$, and assuming $\hat{q}_1 \perp \hat{q}_2$, then we have:

$$\mathrm{MSE}(Y(R, \hat{q})) = (b_1^2 + \nu^2)\mu_1^2 + 2b_1 b_2 \mu_1 \mu_2 + (b_2^2 + \nu^2)\mu_2^2 + \sigma^2 T(T + b_1 + b_2).$$

If $\hat{T} = T$, such as in the case where q and $\hat{q}$ are both normalized to 1 (for example, when estimating risk or prevalence), then

$$\begin{aligned} E[(\hat{q}_1 - q_1)(\hat{q}_2 - q_2) \mid \hat{T} = T] &= E[(\hat{q}_1 - q_1)(T - \hat{q}_1 - T + q_1) \mid \hat{T} = T] \\ &= E[-(\hat{q}_1 - q_1)^2 \mid \hat{T} = T] \leq 0. \end{aligned}$$

If $\mu_1, \mu_2 \geq 0$ the cross term of the MSE is then nonpositive, as would be the case if μ represented a risk or pollution levels.

S.2. Integration grid generation and urbanicity classification

To generate an integration grid over Kenya, we first project the approximate latitudinal and longitudinal extent of Kenya using a projection with European Petroleum Survey Group (EPSG) code 21097, which is accurate to 6m in Kenya [1]. Once the extent is projected into easting/northing (in kilometers) we construct a grid with uniform resolution under the projected coordinate system, removing points outside of Kenya from the integration grid. If certain areas of interest contain no integration points, custom integration points are added at the centroids of those areas. This add flexibility to the integration grid resolution, in particular in the presence of small areas of interest that may not be well represented at a given resolution otherwise.

We first construct a fine scale grid with 1 km resolution for the purpose of estimating urban and rural population at the admin2 level. We assume, as is the case for Kenya, that estimates of general population totals are available at the admin1 level and for the urban and rural portions of each Admin1 area. In the case of Kenya, this is available in the 2009 Kenya Population and Housing Census. Given all spatial locations in the integration grid in a given admin1 area, say $\boldsymbol{s}_1, \ldots, \boldsymbol{s}_L$, with general population densities, $\rho(\boldsymbol{s}_1), \ldots, \rho(\boldsymbol{s}_L)$, we first set a threshold, K, so that,

$$K = \arg\min_k \frac{\sum_{l:\rho(\boldsymbol{s}_l) \geq k} \rho(\boldsymbol{s}_l)}{\sum_l \rho(\boldsymbol{s}_l)} \geq P^{\text{RUR}},$$

where P^{RUR} is the proportion of general population that is rural in the Admin1 area of interest. We then classify location $\boldsymbol{s}_l$ as urban if $q(\boldsymbol{s}_l) \geq K$ for $l = 1, \ldots, L$.

All of this is performed in the SUMMER package in R [2] via the `makePopIntegrationTab` function, using population density data and shapefiles provided on github at https://github.com/paigejo/SUMMERdata.

S.3. Relationship between the aggregation models

S.3.1. Shared expectations

We show in this section that the gridded, empirical, latent, and smooth latent models all have the same central predictions. This is because $E_{\mathbf{Z}}[p_{\text{emp}}(A)] = r_{\text{latent}}(A)$, where $\mathbf{Z} = \{Z_i\}_{i=1}^M$ and the subscript of the expectation denotes what is expected over. Further, $E_{\mathbf{Z},\mathbf{N},\boldsymbol{\epsilon}}[p_{\text{emp}}(A)] = E_{\mathbf{N},\boldsymbol{\epsilon}}[r_{\text{latent}}(A)] = r_{\text{smooth}}(A) = E_{\boldsymbol{\epsilon}}[r_{\text{grid}}(A)]$, where $\mathbf{N} = \{N_i\}_{i=1}^M$ and $\boldsymbol{\epsilon} = \{\epsilon_i\}_{i=1}^M$. For similar reasons, the expectation of the four sampling frame models considered are also the same when applied to burden.

While it is clear that the smooth latent and gridded models use (3) to estimate areal smooth risk and smooth burden choosing a continuously indexed population density q as the spatial density, it is worth noting that the empirical and latent models can also be viewed as using (3), choosing the integrand, $r(\boldsymbol{s})$, to be either the EA level prevalence or risk respectively. Rather than integrating $r(\boldsymbol{s})$ over a continuously indexed spatial density, the empirical and latent models choose $Q(\boldsymbol{s})$ to be a point process for the EA locations and integrate with respect to this point process. Hence, the empirical, latent, and smooth latent sampling frame models are all closely related.

For the latent aggregation model, the areal estimates can be shown to be an expectation over part of the sampling frame model of the empirical areal estimates:

$$
\begin{aligned}
E_{\mathbf{Z}}\left[p_{\text{emp}}(A)\right] &= E_{\mathbf{Z}}\left[\sum_{i:\boldsymbol{s}_i\in A}\frac{N_i}{N}\times\frac{Z_i}{N_i}\right] \\
&= \sum_{i:\boldsymbol{s}_i\in A}\frac{N_i}{N}\times E_{\mathbf{Z}}\left[\frac{Z_i}{N_i}\right] \\
&= \sum_{i:\boldsymbol{s}_i\in A}\frac{N_i}{N}\times r_i \\
&= r_{\text{latent}}(A).
\end{aligned}
$$

Hence, the tower property of expectations implies $E\left[p_{\text{emp}}(A)\right] = E[r_{\text{latent}}(A)]$. Further,

$$
\begin{aligned}
E_{\mathbf{Z},\mathbf{N},\boldsymbol{\epsilon},\{\boldsymbol{s}_i\}_{i=1}^M}\left[p(A)\right] &= E_{\mathbf{Z},\mathbf{N},\boldsymbol{\epsilon},\{\boldsymbol{s}_i\}_{i=1}^M}\left[\sum_{i:\boldsymbol{s}_i\in A}\frac{N_i}{N}\times\frac{Z_i}{N_i}\right] \\
&= E_{\mathbf{N},\{\boldsymbol{s}_i\}_{i=1}^M}\left[\sum_{i:\boldsymbol{s}_i\in A}\frac{N_i}{N}\times E_{\mathbf{Z},\boldsymbol{\epsilon}}\left[\frac{Z_i}{N_i}\right]\right] \\
&= E_{\{\boldsymbol{s}_i\}_{i=1}^M}\left[\sum_{i:\boldsymbol{s}_i\in A}E_{\mathbf{N}}\left[\frac{N_i}{N}\right]\times E_{\boldsymbol{\epsilon}}[r_i]\right] \\
&= E_{\{\boldsymbol{s}_i\}_{i=1}^M}\left[\sum_{i:\boldsymbol{s}_i\in A}Q_A\times r_{\text{smooth}}(\boldsymbol{s}_i)\right] \\
&= \int_A q(\boldsymbol{s})Q_A\times r_{\text{smooth}}(\boldsymbol{s})\,\mathrm{d}\boldsymbol{s}, \\
&= r_{\text{smooth}}(A),
\end{aligned}
$$

where q is proportional to the population density and Q_A is the expected proportion of the total target population in A contained in a single EA in A, so that $\int_A q(\boldsymbol{s}) Q_A \, \mathrm{d}\boldsymbol{s} = 1$. It is easy to see that $E[r_{\text{grid}}(A)] = E[r_{\text{smooth}}(A)]$. Hence, $E[p_{\text{emp}}(A)] = E[r_{\text{latent}}(A)] = E[r_{\text{grid}}(A)] = E[r_{\text{smooth}}(A)]$. A similar argument shows that $E[b_{\text{emp}}(A)] = E[b_{\text{latent}}(A)] = E[b_{\text{grid}}(A)] = E[b_{\text{smooth}}(A)]$.

When producing estimates for an administrative area stratified by urban/rural, one can take a weighted average (or a sum, in the case of burden) of the estimates in the urban and rural parts of that area, weighted by the target population totals in the urban and rural parts of the area.

S.3.2. Relative variances

The law of total variance and some algebra shows that the variance of the empirical aggregation model predictions is necessarily greater than or equal to the variance of the smooth latent predictions when it comes to prevalence and burden. Assuming there is a nonzero target population in area A, we find:

$$\begin{aligned}\text{Var}(p_{\text{emp}}(A)) &= E[\text{Var}_{\boldsymbol{Z},\boldsymbol{N},\boldsymbol{\epsilon},\{\boldsymbol{s}_i\}_{i=1}^M}(p_{\text{emp}}(A))] + \text{Var}(E_{\boldsymbol{Z},\boldsymbol{N},\boldsymbol{\epsilon},\{\boldsymbol{s}_i\}_{i=1}^M}[p_{\text{emp}}(A)]) \\ &= E[\text{Var}_{\boldsymbol{Z},\boldsymbol{N},\boldsymbol{\epsilon},\{\boldsymbol{s}_i\}_{i=1}^M}(p_{\text{emp}}(A))] + \text{Var}(r_{\text{smooth}}(A)).\end{aligned}$$

With similar arguments it is trivial to show,

$$\begin{aligned}&\text{Var}(p_{\text{emp}}(A)) \geq \text{Var}(r_{\text{latent}}(A)) \geq \text{Var}(r_{\text{smooth}}(A)), \quad \text{and} \\ &\text{Var}(b_{\text{emp}}(A)) \geq \text{Var}(b_{\text{latent}}(A)) \geq \text{Var}(b_{\text{smooth}}(A)).\end{aligned}$$

While this argument does not apply directly to the variance of relative prevalence compared with the variance of the relative smooth risk since these are ratios, we find evidence in Section 7 that the variance of relative prevalence is not only larger than the variance of smooth risk, but larger by more a greater factor than for burden and prevalence/risk, provided the relative prevalence and relative burden are defined.

S.3.3. Convergence of the Gridded to the Smooth Latent aggregation model

We will first show that the burden for an area under the Gridded model converges to the Smooth Latent burden. Let $\{G^k : k = 1, 2, \ldots\}$ be aggregation grids of increasing resolution over an area A, so that,

$$b_{\text{grid}}^{(k)}(A) = \sum_{g \in G^{(k)}} r(\boldsymbol{s}_g) q(\boldsymbol{s}_g)$$

is the Gridded estimate of the burden in A based on aggregation grid $G^{(k)}$, where,

$$r(\boldsymbol{s}) = \text{expit}(\boldsymbol{d}^T(\boldsymbol{s})\boldsymbol{\beta} + u(\boldsymbol{s}) + \epsilon(\boldsymbol{s})),$$

for white noise process $\epsilon(\boldsymbol{s}) \overset{iid}{\sim} N(0, \sigma_\epsilon^2)$. We can write,

$$r(\boldsymbol{s}) = \int_{-\infty}^{\infty} \text{expit}(\boldsymbol{d}^T(\boldsymbol{s})\boldsymbol{\beta} + u(\boldsymbol{s}) + \epsilon) p_\epsilon(\epsilon) \, \mathrm{d}\epsilon + \epsilon^*(\boldsymbol{s}),$$

where $E[\epsilon^*(\boldsymbol{s})] = 0$ for all $\boldsymbol{s}$, and $\text{Var}(\epsilon^*(\boldsymbol{s})) \leq \sigma_\epsilon^2 < \infty$ due to the logit link function having derivative magnitude smaller than 1. For other link functions, such as a log link, the variance will also be finite. We also have $\epsilon^*(\boldsymbol{s}) \perp \epsilon^*(\boldsymbol{t})$ for $\boldsymbol{s} \neq \boldsymbol{t}$, which can be verified by writing out $\text{Cov}(\epsilon^*(\boldsymbol{s}), \epsilon^*(\boldsymbol{t}))$ in terms of $r(\boldsymbol{s})$, $r_{\text{smooth}}(\boldsymbol{s})$, $r(\boldsymbol{t})$, and $r_{\text{smooth}}(\boldsymbol{t})$ and applying the law of iterated expectation. Hence,

$$
\begin{aligned}
b_{\text{grid}}^{(k)}(A) &= \sum_{g \in G^{(k)}} r_{\text{smooth}}(\boldsymbol{s}_g) q(\boldsymbol{s}_g) + \sum_{g \in G^{(k)}} \epsilon^*(\boldsymbol{s}_g) q(\boldsymbol{s}_g) \\
b_{\text{grid}}(A) &= \lim_{k \to \infty} \sum_{g \in G^{(k)}} r_{\text{smooth}}(\boldsymbol{s}_g) q(\boldsymbol{s}_g) + \sum_{g \in G^{(k)}} \epsilon^*(\boldsymbol{s}_g) q(\boldsymbol{s}_g) \\
&= \int_A r_{\text{smooth}}(\boldsymbol{s}) q(\boldsymbol{s}) \, \mathrm{d}\boldsymbol{s} + \int_A \epsilon^*(\boldsymbol{s}) q(\boldsymbol{s}) \, \mathrm{d}\boldsymbol{s} \\
&= \int_A r_{\text{smooth}}(\boldsymbol{s}) q(\boldsymbol{s}) \, \mathrm{d}\boldsymbol{s} \\
&= b_{\text{smooth}}(A).
\end{aligned}
$$

Of course, in practice $b_{\text{smooth}}(A)$ is approximated using a gridded sum, so the approximation of $b_{\text{smooth}}(A)$ and $b_{\text{grid}}^{(k)}(A)$ will converge to the true smooth burden $b_{\text{smooth}}(A)$ as the grid resolutions increase, and therefore to each other.

By substituting $\tilde{q}(\boldsymbol{s}) = \frac{q(\boldsymbol{s})}{\int_A q(\boldsymbol{s}) \, \mathrm{d}\boldsymbol{s}}$ for $q(\boldsymbol{s})$, the same proof as above shows that $r_{\text{smooth}}(A)$ and $b_{\text{grid}}^{(k)}(A)$ will converge to the true smooth risk $r_{\text{smooth}}(A)$ as the grid resolutions increase, and therefore to each other.

Provided $P(r_{\text{smooth}}(A^{\text{RUR}}) = 0) = P(r_{\text{grid}}(A^{\text{RUR}}) = 0) = 0$, where A^{RUR} is the rural part of A and $A^{\text{URB}} = A \backslash A^{\text{RUR}}$ is the urban part, then we again have that $r_{\text{smooth}}(A^{\text{URB}})/r_{\text{smooth}}(A^{\text{RUR}})$ and $r_{\text{grid}}^{(k)}(A^{\text{URB}})/r_{\text{grid}}^{(k)}(A^{\text{RUR}})$ will converge to the true relative smooth risk $r_{\text{smooth}}(A^{\text{URB}})/r_{\text{smooth}}(A^{\text{RUR}})$ as the grid resolutions increase.

S.4. Additional grid resolution test results

S.4.1. Grid resolution test results for $\sigma_S^2 = 1/27$

	Model	Units	200m	1km	5km	25km
95% CI width	Empirical	(per 1,000)	9.8 (0.1)	9.8 (0.1)	9.8 (0.1)	9.9 (0.1)
	Latent		8.2 (0.1)	8.2 (0.1)	8.2 (0.1)	8.3 (0.1)
	Smooth Latent		7.7 (0.1)	7.7 (0.1)	7.7 (0.1)	7.7 (0.1)
	Gridded		8.5 (0.1)	19 (0.3)	56.5 (1.0)	60.1 (1.0)
95% CI coverage	Empirical	(Percent)	96 (1.0)	96 (1.0)	96 (1.0)	96 (1.1)
	Latent		91 (1.5)	91 (1.6)	91 (1.5)	91 (1.6)
	Smooth Latent		89 (1.7)	89 (1.7)	89 (1.6)	89 (1.6)
	Gridded		92 (1.4)	100 (0.1)	100 (0.0)	100 (0.0)

Table D.5: 95% credible interval (CI) widths in neonatals per thousand, and empirical coverages in percent for considered sampling frame models as a function of aggregation grid resolution, with $\sigma_S^2 = 1/27$ and standard errors given in parentheses.

	Model	Units	200m	1km	5km	25km
50% CI width	Empirical	(per 1,000)	3.4 (0.0)	3.4 (0.0)	3.4 (0.0)	3.4 (0.0)
	Latent		2.8 (0.0)	2.8 (0.0)	2.8 (0.0)	2.8 (0.0)
	Smooth Latent		2.6 (0.0)	2.6 (0.0)	2.6 (0.0)	2.7 (0.0)
	Gridded		2.9 (0.0)	6.3 (0.1)	16.3 (0.3)	17.5 (0.3)
50% CI coverage	Empirical	(Percent)	50 (2.2)	50 (2.2)	51 (2.3)	50 (2.3)
	Latent		44 (2.1)	44 (2.1)	44 (2.1)	44 (2.1)
	Smooth Latent		41 (2.0)	41 (2.0)	42 (2.1)	42 (2.1)
	Gridded		45 (2.1)	77 (2.0)	99 (0.5)	99 (0.3)

Table D.6: 50% credible interval (CI) widths in neonatals per thousand, and empirical coverages in percent for considered sampling frame models as a function of aggregation grid resolution, with $\sigma_S^2 = 1/27$ and standard errors given in parentheses.

S.4.2. Grid resolution test results for $\sigma_S^2 = 1/9$

	Model	Units	200m	1km	5km	25km
95% CI width	Empirical	(per 1,000)	11.6 (0.3)	11.6 (0.3)	11.6 (0.3)	11.7 (0.3)
	Latent		10.2 (0.3)	10.2 (0.3)	10.2 (0.3)	10.3 (0.3)
	Smooth Latent		9.8 (0.2)	9.8 (0.2)	9.8 (0.2)	9.8 (0.2)
	Gridded		10.5 (0.3)	20.6 (0.6)	59.0 (1.9)	62.7 (2.0)
95% CI coverage	Empirical	(Percent)	96 (1.2)	96 (1.1)	96 (1.1)	96 (1.2)
	Latent		92 (1.5)	92 (1.5)	92 (1.5)	93 (1.5)
	Smooth Latent		91 (1.7)	91 (1.7)	91 (1.7)	91 (1.6)
	Gridded		93 (1.5)	100 (0.1)	100 (0.0)	100 (0.0)

Table D.7: 95% credible interval (CI) widths in neonatals per thousand, and empirical coverages in percent for considered sampling frame models as a function of aggregation grid resolution, with $\sigma_S^2 = 1/9$ and standard errors given in parentheses.

	Model	Units	200m	1km	5km	25km
50% CI width	Empirical	(per 1,000)	4.0 (0.1)	4.0 (0.1)	4.0 (0.1)	4.0 (0.1)
	Latent		3.5 (0.1)	3.5 (0.1)	3.5 (0.1)	3.6 (0.1)
	Smooth Latent		3.4 (0.1)	3.4 (0.1)	3.4 (0.1)	3.4 (0.1)
	Gridded		3.6 (0.1)	6.9 (0.2)	17.0 (0.6)	18.3 (0.6)
50% CI coverage	Empirical	(Percent)	54 (2.5)	54 (2.5)	54 (2.5)	54 (2.5)
	Latent		49 (2.4)	50 (2.4)	50 (2.4)	49 (2.4)
	Smooth Latent		48 (2.4)	48 (2.4)	48 (2.4)	48 (2.4)
	Gridded		51 (2.5)	75 (2.4)	97 (0.9)	99 (0.7)

Table D.8: 50% credible interval (CI) widths in neonatals per thousand, and empirical coverages in percent for considered sampling frame models as a function of aggregation grid resolution, with $\sigma_S^2 = 1/9$ and standard errors given in parentheses.

S.4.3. Grid resolution test results for $\sigma_S^2 = 1/3$

	Model	Units	200m	1km	5km	25km
95% CI width	Empirical	(per 1,000)	14.2 (0.6)	14.2 (0.6)	14.3 (0.6)	14.4 (0.6)
	Latent		13.1 (0.6)	13.0 (0.6)	13.1 (0.6)	13.3 (0.6)
	Smooth Latent		12.6 (0.5)	12.6 (0.5)	12.7 (0.5)	12.8 (0.5)
	Gridded		13.3 (0.6)	23.7 (1.2)	65.6 (3.6)	69.7 (3.9)
95% CI coverage	Empirical	(Percent)	96 (1.2)	96 (1.2)	96 (1.2)	96 (1.1)
	Latent		93 (1.5)	93 (1.5)	93 (1.5)	93 (1.4)
	Smooth Latent		92 (1.6)	92 (1.6)	92 (1.6)	93 (1.5)
	Gridded		93 (1.5)	99 (0.3)	100 (0.0)	100 (0.0)

Table D.9: 95% credible interval (CI) widths in neonatals per thousand, and empirical coverages in percent for considered sampling frame models as a function of aggregation grid resolution, with $\sigma_S^2 = 1/3$ and standard errors given in parentheses.

	Model	Units	200m	1km	5km	25km
50% CI width	Empirical	(per 1,000)	4.9 (0.2)	4.9 (0.2)	4.9 (0.2)	5.0 (0.2)
	Latent		4.5 (0.2)	4.5 (0.2)	4.5 (0.2)	4.5 (0.2)
	Smooth Latent		4.3 (0.2)	4.3 (0.2)	4.4 (0.2)	4.4 (0.2)
	Gridded		4.6 (0.2)	8.0 (0.4)	19.1 (1.1)	20.6 (1.2)
50% CI coverage	Empirical	(Percent)	52 (2.7)	52 (2.7)	53 (2.7)	53 (2.7)
	Latent		49 (2.6)	49 (2.6)	49 (2.7)	48 (2.6)
	Smooth Latent		47 (2.6)	47 (2.6)	48 (2.6)	47 (2.6)
	Gridded		49 (2.6)	71 (2.5)	96 (1.3)	97 (1.1)

Table D.10: 50% credible interval (CI) widths in neonatals per thousand, and empirical coverages in percent for considered sampling frame models as a function of aggregation grid resolution, with $\sigma_S^2 = 1/3$ and standard errors given in parentheses.

S.5. Additional simulation study results

S.5.1. Admin2 × stratum burden

r_{pop}		1/5			1			5		
r_{samp}	β_0 \ φ	1/9	1/3	1	1/9	1/3	1	1/9	1/3	1
3	0	-19.5	-17.2	-15.8	-11.0	-8.2	-6.2	-3.2	-1.7	-1.3
	-4	-11.0	-8.4	-5.9	-3.0	-1.6	-1.1	-0.6	-0.2	-0.1
1	0	-16.5	-14.0	-12.2	-6.9	-4.9	-3.4	-1.4	-0.7	-0.6
	-4	-6.7	-5.2	-3.6	-1.5	-0.9	-0.7	-0.1	-0.1	-0.1
1/3	0	-12.1	-9.7	-8.6	-3.4	-2.6	-2.0	-0.8	-0.2	-0.2
	-4	-2.9	-2.6	-1.9	0.0	-0.3	-0.3	0.1	-0.1	0.0

Table E.11: Mean percent change in CRPS of the empirical aggregation model relative to the smooth latent aggregation model, where the response is Admin2 × stratum burden. Yellow-green values are better, while indigo values are worse. Results most representative of the application in Section 7 are outlined in red.

r_{pop}		1/5			1			5		
r_{samp}	β_0 \ φ	1/9	1/3	1	1/9	1/3	1	1/9	1/3	1
3	0	-75.7	-72.6	-70.3	-59.7	-51.0	-42.9	-25.7	-15.0	-12.7
	-4	-58.3	-50.1	-39.6	-23.2	-14.8	-10.6	-5.4	-2.1	-1.1
1	0	-71.1	-66.6	-62.7	-44.8	-36.2	-28.5	-11.7	-6.0	-5.0
	-4	-40.4	-34.7	-25.7	-9.9	-7.0	-5.2	-0.6	-1.1	-0.8
1/3	0	-60.9	-54.9	-51.9	-25.6	-20.7	-17.5	-4.9	-2.4	-1.7
	-4	-19.4	-17.1	-13.5	-1.4	-1.1	-1.9	0.6	-0.3	0.0

Table E.12: Mean percent change in 95% interval score of the empirical aggregation model relative to the smooth latent aggregation model, where the response is Admin2 × stratum burden. Yellow-green values are better, while indigo values are worse. Results most representative of the application in Section 7 are outlined in red.

S.5.2. Admin2 prevalence

r_{pop}		1/5			1			5		
r_{samp}	β_0 \ φ	1/9	1/3	1	1/9	1/3	1	1/9	1/3	1
3	0	-7.9	-5.0	-3.0	-1.9	-0.8	-0.4	-0.2	-0.1	-0.1
	-4	-8.4	-5.4	-3.1	-1.8	-0.9	-0.5	-0.5	-0.1	0.0
1	0	-5.4	-3.0	-1.5	-0.9	-0.4	-0.2	-0.1	0.0	0.0
	-4	-4.8	-3.3	-1.8	-1.1	-0.5	-0.4	-0.1	-0.1	-0.1
1/3	0	-2.8	-1.4	-1.0	-0.4	-0.2	-0.1	-0.1	0.0	0.0
	-4	-1.8	-1.8	-1.1	-0.1	-0.2	-0.2	0.1	0.0	0.0

Table E.13: Mean percent change in CRPS of the empirical aggregation model relative to the smooth latent aggregation model, where the response is Admin2 prevalence. Yellow-green values are better, while indigo values are worse. Results most representative of the application in Section 7 are outlined in red.

r_{pop}		1/5			1			5		
r_{samp}	β_0 \ φ	1/9	1/3	1	1/9	1/3	1	1/9	1/3	1
3	0	-49.9	-37.3	-24.9	-16.8	-8.8	-5.0	-2.1	-1.2	-0.8
	-4	-50	-37.7	-24.9	-15.4	-8.9	-4.8	-4.2	-1.2	-0.1
1	0	-37.1	-24.7	-14.9	-7.5	-3.8	-2.0	-1.1	-0.2	-0.3
	-4	-30.3	-24.3	-14.5	-6.9	-4.9	-2.8	-0.4	-0.8	-0.4
1/3	0	-20.5	-11.9	-8.4	-2.2	-2.0	-0.8	-0.3	-0.2	-0.1
	-4	-13.0	-11.4	-8.0	-1.5	-0.9	-1.3	0.4	-0.1	-0.1

Table E.14: Mean percent change in 95% interval score of the empirical aggregation model relative to the smooth latent aggregation model, where the response is Admin2 prevalence. Yellow-green values are better, while indigo values are worse. Results most representative of the application in Section 7 are outlined in red.

S.5.3. Admin2 burden

r_{pop}		1/5			1			5		
r_{samp}	β_0 \ φ	1/9	1/3	1	1/9	1/3	1	1/9	1/3	1
3	0	-18.3	-15.7	-14.2	-9.3	-6.5	-4.7	-2.2	-1.0	-0.8
	-4	-9.4	-6.7	-4.3	-2.1	-0.9	-0.6	-0.4	-0.1	0.0
1	0	-15.1	-12.3	-10.3	-5.4	-3.5	-2.3	-0.9	-0.3	-0.3
	-4	-5.4	-3.9	-2.4	-1.1	-0.5	-0.4	-0.1	-0.1	-0.1
1/3	0	-10.5	-8.0	-6.9	-2.4	-1.7	-1.3	-0.5	-0.1	-0.1
	-4	-1.9	-1.8	-1.2	0.1	-0.2	-0.2	0.1	0.0	0.0

Table E.15: Mean percent change in CRPS of the empirical aggregation model relative to the smooth latent aggregation model, where the response is Admin2 burden. Yellow-green values are better, while indigo values are worse. Results most representative of the application in Section 7 are outlined in red.

r_{pop}		1/5			1			5		
r_{samp}	β_0 \ φ	1/9	1/3	1	1/9	1/3	1	1/9	1/3	1
3	0	-74.2	-70.2	-67.5	-54.7	-43.8	-35.2	-18.4	-8.7	-7.8
	-4	-53.2	-43.1	-30.4	-16.0	-8.7	-5.7	-3.9	-1.0	-0.2
1	0	-68.5	-62.8	-58	-37.3	-27.5	-20.3	-6.6	-2.6	-2.4
	-4	-32.9	-27.0	-17.5	-5.9	-4.1	-2.9	0.0	-0.6	-0.5
1/3	0	-56.4	-49.0	-45.3	-17.7	-13.7	-11.4	-2.7	-1.2	-0.5
	-4	-12.1	-11.2	-8.2	0.3	0.0	-1.0	0.7	-0.2	0.0

Table E.16: Mean percent change in 95% interval score of the empirical aggregation model relative to the smooth latent aggregation model, where the response is Admin2 burden. Yellow-green values are better, while indigo values are worse. Results most representative of the application in Section 7 are outlined in red.

S.5.4. Admin2 relative prevalence

r_{samp}	β_0 \ r_{pop} / φ	1/5			1			5		
		1/9	1/3	1	1/9	1/3	1	1/9	1/3	1
3	0	-22.4	-21.2	-19.3	-17.0	-15.9	-13.8	-9.6	-8.4	-6.9
	-4	-27.1	-26.9	-27.2	-19.9	-19.5	-19.2	-11	-10.2	-10.2
1	0	-19.8	-18.6	-16.8	-13.6	-11.9	-10.8	-6.2	-4.9	-4.0
	-4	-23.3	-23.6	-23.6	-14.5	-14.7	-15.2	-6.2	-6.4	-6.6
1/3	0	-15.3	-14.5	-13.5	-8.3	-7.8	-6.6	-3.0	-2.4	-2.3
	-4	-18.9	-19.1	-19.2	-9.3	-9.2	-10.4	-3.0	-3.0	-3.0

Table E.17: Mean percent change in CRPS of the empirical aggregation model relative to the smooth latent aggregation model, where the response is Admin2 relative prevalence. Yellow-green values are better, while indigo values are worse. Results most representative of the application in Section 7 are outlined in red.

r_{samp}	β_0 \ r_{pop} / φ	1/5			1			5		
		1/9	1/3	1	1/9	1/3	1	1/9	1/3	1
3	0	-78.9	-77.3	-74.8	-72.0	-70.0	-64.3	-56.7	-51.8	-42.5
	-4	-79.2	-78.8	-77.3	-73.6	-72.7	-71.8	-56.7	-55.2	-53.8
1	0	-75.8	-74.0	-70.8	-66.0	-62.0	-57.6	-44.1	-37.5	-30.9
	-4	-74.4	-74.7	-72.7	-63.9	-62.7	-63.0	-39.3	-39.7	-38.8
1/3	0	-68.6	-67.4	-64.6	-52.8	-50.1	-45.3	-25.4	-23.2	-21.1
	-4	-65.8	-65.4	-63.8	-47.1	-48.3	-46.9	-20.0	-20.8	-17.8

Table E.18: Mean percent change in 95% interval score of the empirical aggregation model relative to the smooth latent aggregation model, where the response is Admin2 relative prevalence. Yellow-green values are better, while indigo values are worse. Results most representative of the application in Section 7 are outlined in red.

S.5.5. Admin1 prevalence

r_{samp}	r_{pop} / β_0 \ φ	1/5: 1/9	1/5: 1/3	1/5: 1	1: 1/9	1: 1/3	1: 1	5: 1/9	5: 1/3	5: 1
3	0	0.2	0.7	0.4	-0.1	0.0	-0.1	0.0	0.0	0.0
3	-4	-1.5	-0.6	0.1	-0.2	-0.1	0.0	-0.1	0.0	0.0
1	0	-1.0	0.0	0.1	-0.1	0.0	0.0	0.0	0.0	0.0
1	-4	-0.9	-0.4	-0.1	-0.3	-0.1	0.0	-0.1	0.0	0.0
1/3	0	-0.5	-0.1	-0.1	-0.1	0.0	0.0	0.0	0.0	0.0
1/3	-4	-0.3	-0.4	-0.3	0.0	-0.1	0.0	0.0	0.0	0.0

Table E.19: Mean percent change in CRPS of the empirical aggregation model relative to the smooth latent aggregation model, where the response is Admin1 prevalence. Yellow-green values are better, while indigo values are worse. Results most representative of the application in Section 7 are outlined in red.

r_{samp}	r_{pop} / β_0 \ φ	1/5: 1/9	1/5: 1/3	1/5: 1	1: 1/9	1: 1/3	1: 1	5: 1/9	5: 1/3	5: 1
3	0	-0.1	3.4	2.2	-0.8	-0.4	-1.9	-0.3	-0.1	-0.2
3	-4	-13.5	-5.0	0.4	-1.6	-0.7	-0.5	-0.9	-0.2	-0.2
1	0	-5.5	0.5	0.9	-0.1	-0.2	-0.1	-0.2	-0.1	0.0
1	-4	-4.8	-1.2	-1.7	-1.0	-0.7	-0.2	-0.1	-0.2	-0.2
1/3	0	-2.3	0.1	-0.4	-0.1	-0.4	-0.1	-0.1	-0.1	-0.1
1/3	-4	-1.1	-2.2	-1.1	0.2	-0.3	-0.4	0.0	0.0	-0.1

Table E.20: Mean percent change in 95% interval score of the empirical aggregation model relative to the smooth latent aggregation model, where the response is Admin1 prevalence. Yellow-green values are better, while indigo values are worse. Results most representative of the application in Section 7 are outlined in red.

S.5.6. *Admin1 burden*

r_{samp} \ r_{pop}	β_0 \ φ	1/5: 1/9	1/5: 1/3	1/5: 1	1: 1/9	1: 1/3	1: 1	5: 1/9	5: 1/3	5: 1
3	0	0.0	0.3	0.2	-0.1	0.0	-0.1	0.0	0.0	0.0
	-4	-1.1	-0.5	0.1	-0.1	0.0	0.0	-0.1	0.0	0.0
1	0	-0.7	-0.1	0.0	-0.1	0.0	0.0	0.0	0.0	0.0
	-4	-0.5	-0.3	-0.1	-0.2	-0.1	0.0	0.0	0.0	0.0
1/3	0	-0.3	-0.1	-0.1	0.0	0.0	0.0	0.0	0.0	0.0
	-4	-0.1	-0.2	-0.2	0.0	0.0	0.0	0.0	0.0	0.0

Table E.21: Mean percent change in CRPS of the empirical aggregation model relative to the smooth latent aggregation model, where the response is Admin1 burden. Yellow-green values are better, while indigo values are worse. Results most representative of the application in Section 7 are outlined in red.

r_{samp} \ r_{pop}	β_0 \ φ	1/5: 1/9	1/5: 1/3	1/5: 1	1: 1/9	1: 1/3	1: 1	5: 1/9	5: 1/3	5: 1
3	0	-1.8	1.5	1.8	-0.7	-0.5	-1.0	-0.3	0.0	-0.1
	-4	-10.1	-4.6	-0.3	-1.1	-0.5	-0.4	-0.5	-0.1	-0.1
1	0	-3.7	0.0	-0.1	-0.2	-0.2	-0.1	-0.1	0.0	0.0
	-4	-3.2	-1.1	-1.6	-0.3	-0.6	0.0	-0.1	-0.2	-0.2
1/3	0	-1.8	0.1	-0.5	-0.1	-0.2	0.0	-0.1	-0.1	-0.1
	-4	-0.1	-0.9	-0.7	0.2	-0.1	-0.3	0.0	0.0	-0.1

Table E.22: Mean percent change in 95% interval score of the empirical aggregation model relative to the smooth latent aggregation model, where the response is Admin1 burden. Yellow-green values are better, while indigo values are worse. Results most representative of the application in Section 7 are outlined in red.

S.5.7. Admin1 relative prevalence

r_{pop}		1/5			1			5		
r_{samp}	β_0 \ φ	1/9	1/3	1	1/9	1/3	1	1/9	1/3	1
3	0	-12.8	-9.2	-7.5	-3.6	-2.9	-2.3	-0.3	-0.5	-0.5
	-4	-12.9	-12.0	-11.2	-4.6	-3.7	-3.3	0.0	-0.4	-0.8
1	0	-8.2	-6.3	-4.2	-1.6	-0.9	-0.8	-0.2	-0.2	-0.1
	-4	-6.5	-7.3	-6.6	-1.1	-1.3	-1.2	-0.3	0.0	-0.1
1/3	0	-3.1	-2.8	-1.8	-0.3	-0.2	-0.2	0.0	0.0	0.0
	-4	-2.9	-2.7	-3.0	-0.2	-0.6	-0.6	-0.1	0.0	0.0

Table E.23: Mean percent change in CRPS of the empirical aggregation model relative to the smooth latent aggregation model, where the response is Admin1 relative prevalence. Yellow-green values are better, while indigo values are worse. Results most representative of the application in Section 7 are outlined in red.

r_{pop}		1/5			1			5		
r_{samp}	β_0 \ φ	1/9	1/3	1	1/9	1/3	1	1/9	1/3	1
3	0	-64.1	-54.1	-46.7	-29.7	-23.2	-18.6	-4.6	-4.3	-4.3
	-4	-63.5	-61.5	-60.4	-33.3	-29.5	-27.3	-2.3	-3.1	-5.8
1	0	-49.5	-43.0	-33.2	-13.7	-7.8	-8.5	-1.4	-1.8	-1.3
	-4	-43.5	-48.1	-43.3	-10.3	-10.9	-9.4	-2.4	0.0	0.0
1/3	0	-24.9	-21.7	-15.8	-3.0	-2.2	-1.8	-0.3	-0.3	-0.7
	-4	-19.3	-20.9	-22.9	-1.2	-3.9	-4.0	-0.4	0.2	0.8

Table E.24: Mean percent change in 95% interval score of the empirical aggregation model relative to the smooth latent aggregation model, where the response is Admin1 relative prevalence. Yellow-green values are better, while indigo values are worse. Results most representative of the application in Section 7 are outlined in red.

S.6. Additional NMR application results

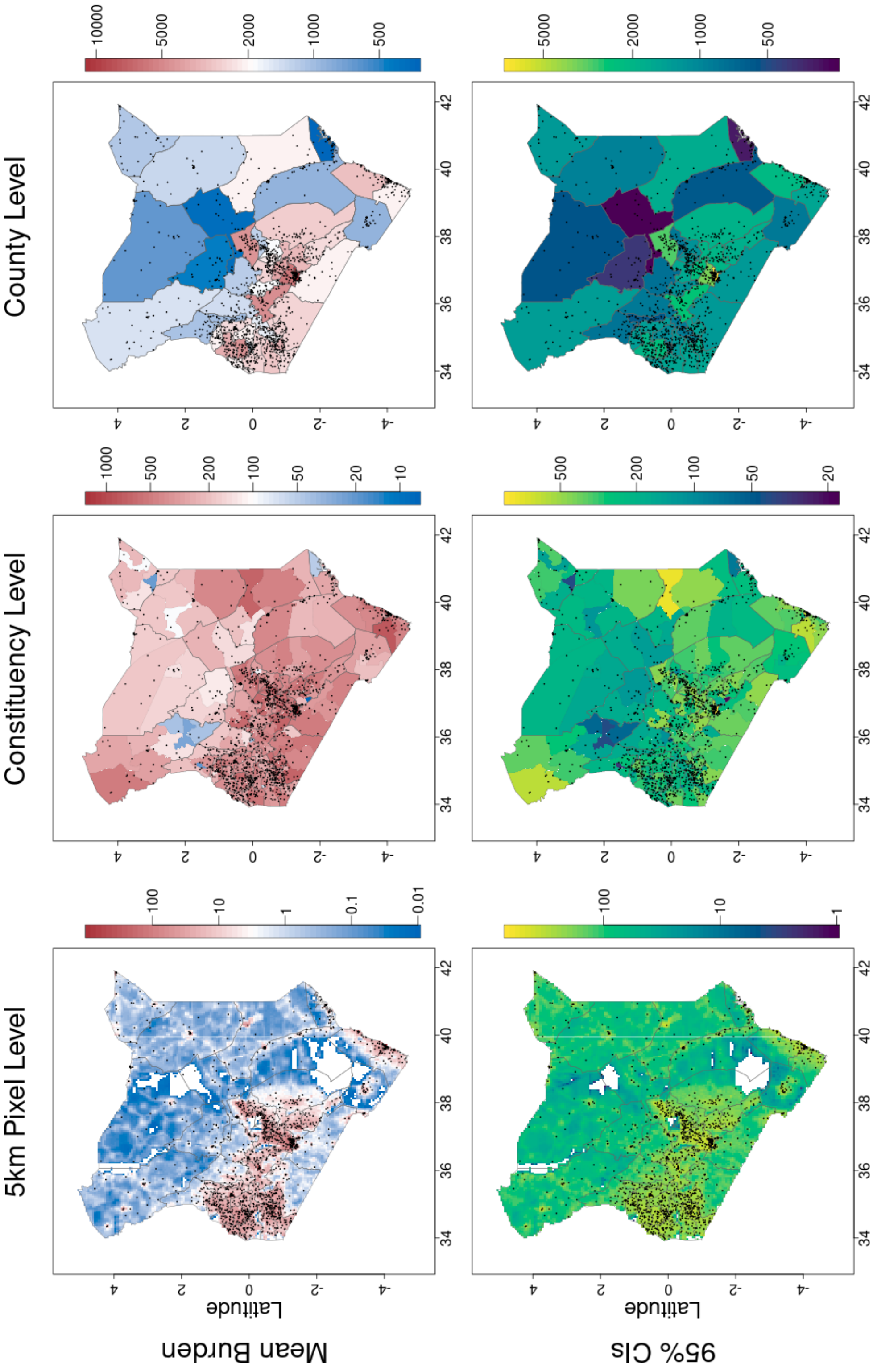

Figure F.3: Central predictions (top row) and 95% credible interval (CI) widths (bottom row) of neonatal mortality burden in Kenya in 2010–2014. Observation locations are plotted as black dots, provinces as thick black lines, and counties as thin grey lines.

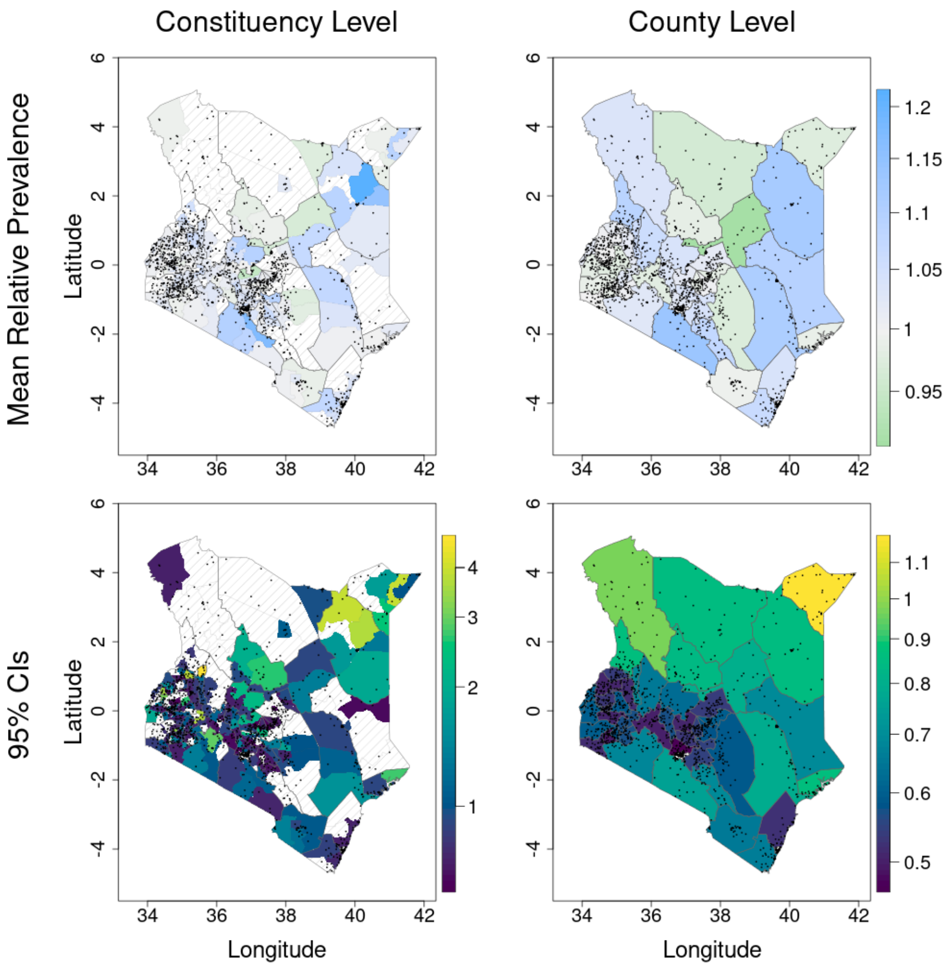

Figure F.4: Central predictions (top row) and 95% credible interval widths (bottom row) of neonatal mortality relative prevalence of urban versus rural populations in Kenya in 2010–2014. Observation locations are plotted as black dots, provinces as thick black lines, and counties as thin grey lines. Crosshatched areas do not have both urban and rural populations and so do not have defined relative prevalence.

S.7. Additional sensitivity analysis results

References

[1] EPSG (2014). EPSG:21097. https://epsg.io/21097-1284.

[2] Li, Z. R., Martin, B. D., Hsiao, Y., Godwin, J., Paige, J., Wakefield, J., Clark, S. J., Fuglstad, G.-A., and Riebler, A. (2021). *SUMMER: Small-Area-Estimation Unit/Area Models and Methods for Estimation in R*. R package version 1.2.0.

Scenario	Area level	*Mean* All models	*Standard deviation* Smooth latent	Latent	Empirical
Meta	Constituency × stratum	98.4	96.6	59.6	54.7
	Constituency	24.4	24.8	22.9	22.1
	County	0.9	2.7	2.6	2.4
2019 census	Constituency × stratum	64.4	65.1	53.3	49.4
	Constituency	38.4	39.8	37.5	36.1
	County	38.3	39.0	38.7	38.2
Jittered census	Constituency × stratum	0.4	2.3	2.3	2.2
	Constituency	0.4	2.4	2.3	2.2
	County	0.4	2.4	2.5	2.4

Table G.25: Mean absolute percent difference of burden posterior mean and SD in sensitivity analysis scenarios compared to with the original data.

Scenario	Area level	*Mean* All models	*Standard deviation* Smooth latent	Latent	Empirical
Meta	Constituency × stratum	–	–	–	–
	Constituency	1.2	6.1	18.3	26.1
	County	3.6	10.8	9.7	8.4
2019 census	Constituency × stratum	–	–	–	–
	Constituency	0.9	3.0	13.2	22.5
	County	1.2	4.4	5.0	7.4
Jittered census	Constituency × stratum	–	–	–	–
	Constituency	0.3	6.3	4.0	3.0
	County	0.4	5.7	4.8	3.8

Table G.26: Mean absolute percent difference of relative prevalence posterior mean and SD in sensitivity analysis scenarios compared to with the original data.
